# Nut consumption and disability-free survival in community-dwelling older adults: a prospective cohort study

**DOI:** 10.1093/ageing/afae239

**Published:** 2024-11-18

**Authors:** Holly Wild, Madina Nurgozhina, Danijela Gasevic, Alison M Coates, Robyn L Woods, Joanne Ryan, Lawrence Beilin, Thara Govindaraju, John J McNeil, Alice J Owen

**Affiliations:** School of Public Health and Preventive Medicine, 553 St Kilda Road Melbourne, 3004 VIC, Australia; School of Public Health and Preventive Medicine, 553 St Kilda Road Melbourne, 3004 VIC, Australia; School of Public Health and Preventive Medicine, 553 St Kilda Road Melbourne, 3004 VIC, Australia; Usher Institute, The University of Edinburgh, 5 Little France Rd, Edinburgh EH16 4UX, UK; Allied Health & Human Performance, University of South Australia, North Terrace, Adelaide SA 5000, Australia; School of Public Health and Preventive Medicine, 553 St Kilda Road Melbourne, 3004 VIC, Australia; School of Public Health and Preventive Medicine, 553 St Kilda Road Melbourne, 3004 VIC, Australia; Medical School, University of Western Australia, 17 Monash Ave, Nedlands WA 6009, Australia; School of Public Health and Preventive Medicine, 553 St Kilda Road Melbourne, 3004 VIC, Australia; School of Public Health and Preventive Medicine, 553 St Kilda Road Melbourne, 3004 VIC, Australia; School of Public Health and Preventive Medicine, 553 St Kilda Road Melbourne, 3004 VIC, Australia

**Keywords:** nut consumption, disability-free survival, older adults, health-span, older people

## Abstract

**Objectives:**

The relationship between nut intake and disability-free survival (healthy lifespan) in later life is unclear. The objective was to evaluate the association between nut intake and disability-free survival in a cohort of adults aged ≥70 years, and whether this varied according to overall diet quality.

**Methods:**

This prospective cohort study involved 9916 participants from the ASPREE Longitudinal Study of Older Persons. Participants completed a 49-item Food Frequency questionnaire from which frequency of nut intake was obtained and were asked to categories usual intake as no/infrequent [never/rarely, 1–2 times/month], weekly [1–2 times/week, often 3–6 times/week] or daily [every day or several times a day]. The outcome measured was a composite of first-event mortality, onset of dementia, or persistent physical disability. Cox proportional hazards regression models, adjusted for socio-demographic factors, health-related and clinical covariates and overall dietary quality were conducted to examine the association between varying levels of nut intake and disability-free survival.

**Results:**

Over a mean of 3.9 years of follow-up, the risk of reaching the DFS endpoint were 23% lower (HR 0.77 [0.61–0.98]) for those who consumed nuts daily, when compared to those with no/infrequent nut consumption. Subgroup analysis demonstrated a significant association between daily nut consumption and healthy lifespan among individuals in the second dietary quality tertile (HR 0.71[0.51–0.98]).

**Conclusion:**

For community-dwelling adults aged 70 years and over with sub-optimal diets, daily nut consumption is associated with the promotion of healthy lifespan (disability-free survival).

## Key Points

- Nuts are a good source of protein, micronutrients unsaturated fats and energy. In adults aged over 70 years, daily nut consumption is associated with promotion of a healthy lifespan, and may be most beneficial for those with sub-optimal dietary quality.

## Introduction

Countries across the world are facing population ageing. [1]. Later life ageing is a complex and multifactorial process that is associated with increased risk of morbidity [2]. After the fourth decade of life there is generally a gradual loss of lean muscle mass, bone mass and geometry, and redistribution of fat that impact the function and strength of the musculoskeletal system and leads to a higher risk of accidental falls, fractures, disability and mortality [3]. Alongside physical changes, cognitive decline is also common in older age. Age-related increases in oxidative stress, inflammation and vascular impairment can lead to neuron death and synapse loss, increasing the risk of dementia [4]. Dietary intake is an important modifiable risk factor for both physical and cognitive decline, and to reduce the risk of chronic disease [5].

Nuts contain a variety of vitamins (folate, niacin, vitamin E), minerals (selenium, magnesium, calcium, potassium), and are high in fiber, polyphenols, phytosterols, and monounsaturated and polyunsaturated fats [6]. Nuts also provide a good source of protein, which is essential for muscle maintenance in older age [3], pistachios and peanuts in particular having a higher amount of free amino acids than other plant-based foods [7]. The benefits of this nutritional profile are supported by a growing evidence suggesting that frequent nut consumption reduces the risk of cardiometabolic conditions [8, 9], cancer [10, 11], dementia [12] and mortality [13]. The energy density and nutrient density of nuts makes them a potentially important inclusion in the diets of older adults in order to delay age-related physical [14] and cognitive decline [15].

Nut consumers are often reported to be generally more health conscious than their counterparts who do not consume nuts, reporting higher overall diet quality scores [16, 17], and higher levels of physical activity and health-promoting behaviors and attitudes [16]. However, recent research has also suggested that this relationship may be bi-directional, with the inclusion of nuts in the diet leading to reduced consumption of discretionary foods, and improving overall dietary score [18, 19].

The current evidence base of observational studies on the association between nuts and chronic disease is dominated by studies in middle-aged adults [13], while longitudinal studies have largely focused on specific disease outcomes, such as CVD or cancer [9, 13, 20]. This study examines whether nut consumption is associated with a measure of health span, (disability-free survival), in adults aged 70 years and over.

## Materials and methods

### Study population

The present post-hoc analysis used data collected during the ASPREE (ASPirin in reducing Events in the Elderly) trial and one of its major sub-studies. ASPREE was a double-blind, placebo-controlled randomized clinical trial that investigated the effect of daily intake of 100 mg enteric-coated aspirin on the extension of healthy life in 19,114 older adults in Australia and the US [21, 22]. Participants were aged 70+ years, except US minority participants who were aged 65+ years, and were pre-screened to be free of dementia, cardiovascular disease events and major physical disabilities [21]. Inclusion and exclusion criteria for ASPREE have been previously described [21, 22]. The ASPREE Longitudinal Study of Older Persons (ALSOP) was a sub-study of ASPREE in Australian participants that investigated social, behavioral and other factors related to healthy ageing, to which over 85% of Australian ASPREE participants contributed [23]. The present study uses exposure data collected from the ALSOP study, and thus includes on Australian participants aged over 70 years.

### Exposure variable: nut consumption

Nut consumption was assessed at Year 3 of ALSOP as part of a self-administered 49-item food frequency questionnaire (see Appendix 1 in Supplementary Data for the Food Frequency Questionnaire) [23], and this timepoint is taken as the origin for this analysis. Participants were asked ‘*how often over the past 12 months did you eat nuts?* with a frequency scale given ranging from no/infrequent consumption [*never/rarely” to “once or twice/month*], weekly consumption [“*once or twice/week to “often 3–6 times/week*] and daily consumption [*every day or several times a day*]. The type and form of nut (i.e. whole or paste, roasted or raw) were not distinguished, so the response is interpreted as representing total nut intake.

### Assessment/ascertainment of disability-free survival

The primary outcome was disability-free survival, which is a composite measure of survival without dementia or major physical disability and was assessed as the first event of death, or onset of dementia or persistent loss of one of six basic activities of daily living (persistent physical disability) or admission to a nursing care facility for a physical disability if the Katz ADL questionnaire could not be administered. This adjudicated outcome has been explained in detail previously [24] Mortality data was obtained from medical records or close contact notifications and confirmed with a second independent source, such as a primary care provider. Linkage to the Australian death registry (National Death Index) was also undertaken [21, 22]. The diagnosis of dementia was made according to the Diagnostic and Statistical Manual (DSM) IV criteria. Physical disability was established when a participant self-reported at an annual visit or six monthly phone calls, having a major difficulty with or unable to perform or needing assistance with at least one of six basic activities including bathing, dressing, toileting, transferring from chair or bed, walking across a room and feeding, that persisted for at least 6 months, as previously described [25].

### Assessment of other covariates/variables

Demographic and socio-economic characteristics including age, gender (women/men), education level (12 years or less or more than 12 years), smoking status (never/former/current), alcohol use (non-consumption, consumption within recommended guidelines, or above recommended intake (*consumption of no more than 10 standard drinks per week, and no more than four drinks on any occasion*), [26] and area-level socioeconomic status (the Index of Relative Socio-economic Advantage and Disadvantage (IRSAD) quintiles) [27] were included as covariates in this analysis. As habitual physical activity data was not available, as a proxy, physical ability was assessed by participants’ self-report of being able to walk for more than 15 min without needing to rest, in the past two weeks.

An overall dietary quality score was generated from dietary information collected in the Year 3 ALSOP medical questionnaire, which included the frequency of intake of common foods based on key food groups and beverages and was scored against the Australian guidelines for healthy eating (see Appendix 1 in Supplementary Data for the Food Frequency Questionnaire). This score was categorized into tertiles.

Biomedical factors were assessed via a combination of clinical and self-reported information at year-3 by the ASPREE follow-up questionnaire (hypertension, type 2 diabetes, frailty and waist circumference) and ALSOP medical questionnaire (oral health status). Cardiometabolic comorbidities were classified as follows, type 2 diabetes, (self-reported or taking prescription medication for diabetes or a fasting glucose ≥126 mg/dL/≥7 mmo/L), and hypertension (systolic blood pressure ≥ 140 mmHg and/or diastolic blood pressure ≥ 90 mmHg or taking blood pressure-lowering medication), and were collected annually [21, 22]. Frailty was measured via a 67-item frailty index, each component of which has been described previously [28]. The frailty index was calculated as the cumulative score across all deficits divided by the number of deficits included. Participants were classified as non-frail ≤0.10), pre-frail (<0.10 and ≤ 0.21), or frail (>0.21) [28]. Depression symptoms were assessed using the Center for Epidemiologic Studies Short Depression Scale (CES-D-10) [29]. A score of less than 2 signifies no depressive symptoms, between 3–7 mild depressive symptoms and 8 or above indicates significant depressive symptoms.

### Statistical analysis

Participant characteristics were presented as counts and percentages based on the level of nut consumption. Differences in study characteristics between these levels of consumption were assessed using Chi-square tests. We performed correlation analyses between the hypothesized correlates of nut consumption to examine potential collinearity. The highest correlation coefficient reported was between nut consumption and dietary score (r = 0.30).

Cox proportional hazards regression analysis was used to assess the association of nut intake and disability-free survival. A crude model was performed followed by; a minimally adjusted model [age and sex], and the fully adjusted model [age, sex IRSAD quintile and education, physical capacity, smoking status and alcohol consumption, waist circumference, hypertension, type 2 diabetes, depression score, frailty status, and self-reported oral health status and dietary quality score]. As overall diet quality has been consistently demonstrated as a moderator in observational nutrition research [30], we also performed a sub-group analysis stratified by dietary score tertile, to better understand the influence of dietary quality on the association between nuts intake and DFS. All variables were tested for interaction within the model. A sensitivity analysis controlled for total protein diversity was performed to assess the influence of total protein intake (red meat, poultry, dairy, eggs, pulses and fish) on the association between nuts and DFS (see Appendix 2 in Supplementary Data for sensitivity analysis methodology).

Statistical significance was set at p-value<0.05 and 95% CIs were calculated for all HR’s. Statistical analysis was performed using STATA software, version 17.0 (Stata Corp., College Station, TX, USA).

## Results

### Nut consumption and baseline characteristics

**Figure 1:**
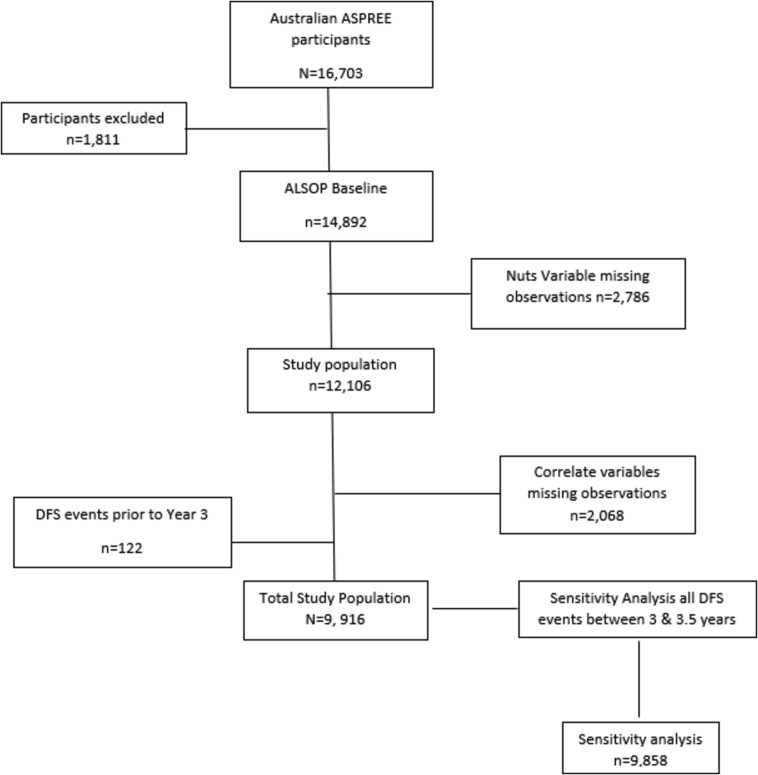
Participant Flowchart.

The removal of those who accrued the composite endpoint prior to the Year 3 assessment of nut intake (n = 112), resulted in a study population of 9916 participants (Figure 1), consisting of 5352 (54%) women and 4564 (46%) men, with a mean age of the population of 77 ± 4.1 years. No interactions were identified within the primary model.

Participants who consumed nuts daily, were more likely to be women, to be younger, to have a lower waist circumference, and to reside in higher socioeconomic status areas. Additionally, they demonstrated higher physical ability, were more likely to have never smoked, to report excellent oral health and to be in the highest dietary quality tertile. Those who consumed nuts daily were also less likely to be frail or have diabetes, but were more likely to exceed the alcohol guidelines (Table 1).

**Table 1:** Baseline characteristics of participants according to the frequency of nut intake

	Total (N = 9, 916)	No/Infrequently (n = 4803) 48.4%	Weekly (n = 3978) 40.1%	Daily (n = 1135) 11.5%	*P* value
Gender					0.002
Female	5352 (54.0)	2538 (52.8)	2149 (54.0)	655 (58.6)	
Male	4564 (46.0)	2265 (47.2)	1829 (46.0)	470 (41.4)	
Age mean (SD)	77.50 (4.8)	77.70 (4.1)	77.30 (4.0)	77.40 (4.1)	<0.001
Age					<0.001
70–74.99	2860 (28.8)	1293 (26.9)	1223 (30.8)	341 (30.0)	
75–79.99	4465 (45.1)	2190 (45.6)	1775 (44.6)	500 (44.1)	
80–84.99	1866 (18.8)	932 (19.4)	731 (18.4)	203 (17.9)	
85+	725 (7.3)	338 (8.1)	246 (6.2)	91 (8.0)	
IRSAD quintile^*a*^					<0.001
1	1572 (15.8)	869 (18.1)	563 (14.1)	140 (12.3)	
2	1640 (16.5)	844 (17.6)	623 (15.7)	173 (15.3)	
3	1831 (18.5)	922 (19.2)	717 (18.0)	192 (16.9)	
4	1943 (19.6)	928 (19.3)	791 (19.9)	224 (19.7)	
5	2903 (29.6)	1240 (25.8)	1284 (32.3)	406 (35.8)	
Education					<0.001
</= 12 years	5817 (58.7)	3109 (64.7)	2153 (54.1)	555 (48.9)	
> 12 years	4099 (41.3)	1694 (35.3)	1825 (45.9)	580 (51.1)	
Physical Activity					<0.001
Waling = <15 min	1418 (14.3)	802(16.7)	502 (12.6)	114 (10.0)	
Walking >15 min	8498 (85.7)	4001 (83.3)	3476 (87.4)	1021 (89.0)	
Smoking					<0.001
Never	5636 (56.8)	2617 (54.5)	2339 (58.8)	680 (59.9)	
Former	3991 (40.3)	2009 (41.8)	1553 (39.0)	429 (37.8)	
Current	289 (2.9)	177 (3.7)	86 (2.2)	26 (2.3)	
Alcohol					<0.001
Non Drinker	2536 (25.6)	1312 (27.3)	916 (23.0)	308 (24.2)	
Meets Guidelines	3895(39.3)	916 (39.4)	1613 (40.6)	402 (35.4)	
Exceeds Guidelines	3485 (35.1)	1611(33.5)	1449 (36.4)	425 (37.4)	
Waist Circ Mean (SD)	96.1 (12.6)	96.9 (12.7)	95.8 (12.5)	93.4 (11.9)	<0.001
Hypertension					<0.001
No	1323 (13.3)	561 (11.7)	563 (14.2)	199 (17.5)	
Yes	8593 (86.7)	4242 (88.3)	3415 (85.8)	936 (82.5)	
Diabetes					0.034
No	8760 (88.3)	4210 (87.8)	3525 (88.6)	1025 (90.3)	
Yes	1156 (11.7)	593 (12.2)	453 (11.4)	110 (9.7)	
Frailty Score					<0.001
Non frail	5, 162 (52.1)	2280 (44.5)	2202 (55.4)	680 (59.9)	
Pre-Frail	3, 731 (37.6)	1935 (40.3)	1414 (35.6)	382 (33.7)	
Frail 2	1023 (10.3)	588 (12.2)	362 (9.1)	73 (6.4)	
Self-reported oral health					<0.001
Poor	96 (0.9)	63 (1.3)	26 (0.6)	7 (0.6)	
Fair good	4311 (43.5)	2234 (46.5)	1658 (41.7)	419 (36.9)	
V.good excellent	5509 (55.6)	1124 (52.2)	2294 (57.7)	709 (62.5)	
Depression^*b*^					0.021
None	2873 (29.0)	1380 (28.7)	1161 (29.2)	332 (29.2)	
Mild	5476 (55.2)	2606 (54.3)	2222 (55.9)	648 (57.1)	
Mild to moderate	1567 (15.8)	817 (17.0)	595 (14.9)	155 (13.7)	
Diet Score					<0.001
T1 – Low	379 (3.8)	305 (6.3)	69 (1.7)	5 (0.5)	
T2 – Moderate	6799 (68.6)	3606 (75.1	2648 (66.6)	545 (48.0)	
T3 – High	2738 (27.6)	892 (18.6)	1261 (31.7)	585 (51.5)	

*Values given as n(%) except for age, waist circumference (Mean, SD)*

a *Index of Relative Socio-economic Advantage and Disadvantage, Socioeconomic Index for Areas, Australian Bureau of Statistics 2012* [25–27]

b *Depression symptoms assessed using the CESD* [28, 29]

### Outcome: Nut consumption and disability-free survival

**Figure 2:**
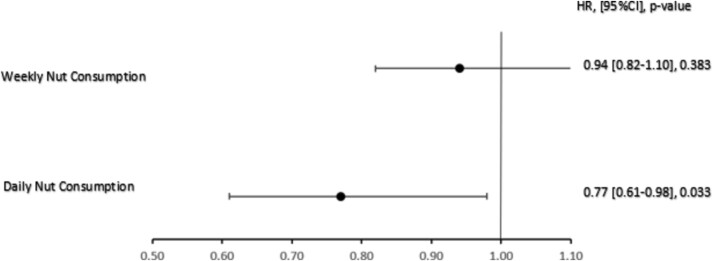
Fully Adjusted Cox Proportional Hazard Ratio Model, HR [95%CI].

During the 3.9 years of follow-up, a total of 997 composite endpoints were recorded, 536 (63.8%) were reported in men and 461 (46.2%) in women (Table 2). In the fully adjusted Cox proportional hazards regression model, daily nut consumption exhibited a significant association with DFS. The risk of reaching the DFS endpoint were 23% lower (HR 0.77 [0.61–0.98]) for those who consumed nuts daily when compared to those who with no/infrequent consumption (Table 3. Figure 2). Subgroup analysis demonstrated a significant association between daily nut consumption and healthy lifespan among individuals in the second dietary tertile. Daily consumption was associated with a lower risk of reaching the DFS endpoint by 29% (HR 0.71[0.51–0.98], *P =* 0.037) compared to those with no/infrequent consumption. No significant association was demonstrated for daily consumption in the highest or lowest dietary score tertiles (Appendix 2. Table 1 in Supplementary Data 1). The significantly lower risk of reaching the disability-free survival endpoint associated with daily consumption of nuts was maintained in the sensitivity analysis, when additionally controlled for total protein rich food consumption (Appendix 2. Table 2 in Supplementary Data).

**Table 2:** Disability free survival endpoint *^a^**by nut consumption*

Outcome	Outcome N = 9, 916	No/Infrequently (n = 4803) 48.4%	Weekly (n = 3978) 40.1%	Daily (n = 1135) 11.5%	*P* value
DSF					<0.001
No	8919 (89.9)	4255 (89.6)	3615 (90.9)	1049 (92.4)	
Yes	997 (10.1)	548 (11.4)	363 (9.1)	89 (7.6)	
Men					
No	4028 (88.3)	1961 (86.6)	1635 (90.4)	432 (91.9)	<0.001
Yes	536 (11.7)	304 (13.4)	194 (10.6)	38 (8.1)	
Women					
No	4891 (91.4)	2294 (90.4)	1980 (92.1)	617 (92.8)	
Yes	461 (8.6)	244 (9.6)	169 (7.9)	48 (7.2)	

a *Disability-free survival composite endpoint reached, representing occurrence of first event of dementia or persistent physical disability or mortality.*

**Table 3:** *Cox proportional hazards regression analysis of the association between nut consumption and* risk of composite DFS endpoint^*c*^

Crude Model	
Nut Consumption	HR [95%CI’s], p-value
No/Infrequent	Ref
Weekly	0.79 [0.69–0.91], 0.001
Daily	0.65 [0.52–0.82]. <0.001
Minimally Adjusted Multivariate Model^*a*^	
No/Infrequent	Ref
Weekly	0.86 [0.75–0.99] 0.026
Daily	0.68 [0.55–0.90], 0.001
Fully Adjusted Multivariate Model^*b*^	
No/Infrequent	Ref
Weekly	0.94 [0.82–1.10], 0.383
Daily	0.77 [0.61–0.98], 0.033

a **Minimally Adjusted Model:** Adjusted for Age and Sex

b **Fully Adjusted Model:** Adjusted for IRSAD, education physical ability, smoking status, alcohol consumption, waist circumference hypertension, type 2 diabetes, depression (CES-D-10), frailty score, self-reported oral health & diet quality score tertile.

c **DFS** = Disability, Dementia or Death

## Discussion

In this prospective cohort study of adults aged 70 years and over, we found that daily nut consumption was associated with healthy lifespan, after adjustment of potential confounding factors, which was particularly evident in those with lower-than-optimal diet quality. To the best of our knowledge, the association between frequent nut consumption and healthy lifespan, as a composite of all-cause mortality, dementia and persistent physical disability, has not previously been reported.

Previous studies have reported inverse association between nut consumption and all-cause mortality [11, 31, 32]. In a recent prospective cohort study of men aged ≥35 years [31], high, compared to low, nut consumption was associated with a 15% lower risk of death (HR 0.85; [95% CI: 0.75–0.96]). In a recent meta-analysis of sixteen studies spanning middle-aged to older cohorts, high versus low intake of nuts was associated with a 19% reduction in risk of all-cause mortality (RR 0.81 [95%CI 0.72–0.84]) [11]. Research in older aged cohorts (65 years of age and over) have demonstrated that when compared to rare consumption, three or more servings of nuts weekly may lower the risk of CVD mortality [8], cancer mortality [20] and all-cause mortality [11]. In a study reporting on findings in people with diabetes from both the Nurses’ Health Study (34-year follow-up) and the Health Professionals Follow-Up Study (28-year follow-up) five or more servings of nuts weekly was associated with a lower risk of CVD and all-cause mortality by 34% (HR 0.66 [0.52–0.84]) and 31% (HR 0.69 [0.61–0.77]), respectively [32].

In a recent systematic review [15], two out of four papers examining a population aged 70 years or over reported the potential protective effect of nuts on the cognitive function in women [33]. Regular nut consumption was associated with a 17% lower risk of cognitive decline (RR = 0.83, [0.75–0.91]) among a Chinese elder population who consumed nuts frequently [34].

Recent observational research has demonstrated that long-term reductions in dietary intake and diversity are associated with frailty in older adults, especially for women [35], and that high protein intake in older age was associated with a significantly lower risk of frailty [36]. Nuts are a good source of plant protein, and a recent US based longitudinal study of older women demonstrated a significant, inverse association between frequent (5 or more serves/week) nut intake and frailty, compared to infrequent consumption (1 serve/month) [37].

A prospective study from the ENRICA cohort, demonstrated a significantly lower risk of impaired agility and mobility in men (HR 0.59 [0.39–0.90]; HR 0.50 [0.29–0.90]), and a lower risk of impaired overall physical function in women (HR 0.65 [0.48–0.87]) with higher nut consumption [14]. The relatively high energy and nutrient density of nuts may assist older adults in meeting protein intake recommendations, which in Australia are 25% higher for adults aged over 70 years compared to younger adults, to support maintenance of muscle mass [38], although it is noted that there are some characteristics of whole nuts which may be a barrier to intake in some older adults, especially those with dental issues [39]. The results of our sensitivity analysis suggest that nutritional components beyond protein may also be beneficial to the extension of healthy lifespan.

A key feature of the disability-free survival outcome used in our study is that it represents a functional measure of relevance to maintaining a healthy independent life in older age. Nut intake has been consistently associated with a lower risk of CVD [8, 40, 41] cognitive decline [15, 33, 34] and mortality [8]. The nutritional profile of nuts may provide insights into the shared pathways responsible for these findings. Nuts are a source of polyunsaturated fatty acids, which have been observed to elicit cardio-protective effects, improving endothelial function [42], and reducing inflammation and oxidative stress via promotion of nitric oxide (NO) synthesis. [43] This anti-inflammatory pathway is also supported by the amino acid L-arginine, a precursor to NO, of which nuts are an important source. [42] Unsaturated fatty acids are essential for neuronal membrane integrity [44] and higher hippocampal NO as a result of L-arginine intake, is associated with a lower risk of Alzheimer’s [45]. Phyto-chemicals present in nuts, such as *a*-tocopherols are potent anti-oxidants and have been demonstrated to reduce lipoprotein oxidation and reduce atherosclerosis severity [42]. Nuts are also a good source of soluble and insoluble dietary fiber. [46] Insoluble fiber has pre-biotic effects, enhancing butyrate production in the gut, which may have numerous health-promoting effects, from increased satiety to glucose regulation [47]. Soluble fiber improves bile secretion and may be one of the mechanisms responsible for the cholesterol-lowering effect of nut consumption [47].

Anticipating the association between dietary quality and nut consumption, demonstrated in previous research [15–19], in this analysis we treated diet quality as a moderator, performing sub-group analysis stratified by tertiles of dietary quality score. The results indicated that the significant association between daily nut consumption is moderated by both high (T3) and low (T1) overall dietary quality, but was maintained for daily nut consumers within the middle tertile of dietary quality (T2), suggesting that the addition of nuts can improve healthy lifespan in diets where the daily intake of other health-promoting foods is moderate. This result aligns with an emerging body of research from across the lifespan, that demonstrates the inclusion of daily nuts improves overall nutritional status, health outcomes and nutritional status in diets that are of a moderate quality [17, 48, 49].

The question arises as to whether the association between nut consumption and disability survival described here is causal. Those eating any nuts on a regular basis showed strikingly better lifestyle, demographic and physical than those eating nuts never or rarely. Although we have attempted to correct for a range of confounders it is likely that these effects were underestimated, and/or that other effects were undetected. There is still a possibility that nut intake is associated with increased disability free survival because it is a marker of longstanding lifestyle and demographic factors promoting healthy longevity. This does not exclude some contribution of frequent nut intake on health, and the potential benefits of including nut consumption alongside other clinical recommendations to lower chronic diseases risk.

The major strengths of this study include the large sample size of the cohort and minimal loss to follow-up of the cohort. The ascertainment of death was confirmed in two steps which minimized measurement bias. Subgroup analysis provides an indication of the moderating effect of overall diet quality on the association, and reducing the likelihood that our results are confounded by the influence of overall dietary quality. A limitation is that nut consumption was self-reported, increasing the risk for recall bias. Furthermore, nut intake was not differentiated to nut type and the quantity of nut intake was not captured by the questionnaire. While the current body of research identifies that nuts have differing nutritional compositions [41], meta-analyses of observational studies on the association of nut intake and health outcomes [8, 15] report that heterogeneity within the evidence base limits our understanding of the influence of different types of nuts [8, 15]. The study population is a cohort of older Australians who were independently living and free of chronic disabling disease when they enrolled in the ASPREE clinical trial. As previously reported [23], this cohort had less ethnic diversity than the population from which they were drawn, and results may not be generalizable to the wider older adult population. While the opportunity to explore genetic factors influencing the relationship between dietary protein and health outcomes represents an important avenue for future research [50], it is acknowledged that much of the underpinning evidence from genome-wide association studies is derived from those of European ancestry.

## Conclusion

In conclusion, the findings of our study suggest that daily nut consumption is associated with an improved healthy lifespan in older adults, including in those whose diet quality may not be optimal.

## Supplementary Material

aa-24-0477-File002_afae239

aa-24-0477-File003_afae239

aa-24-0477-File004_afae239

## Data Transparency and Availability

Access to ASPREE and ALSOP data is available via application. Details can be found at: https://ams.aspree.org/public/.

## References

[1] Officer A , Thiyagarajan JA, Schneiders ML et al. Ageism, healthy life expectancy and population ageing: how are they related? Int J Environ Res Public Health 2020; 17: 3159. 32370093 10.3390/ijerph17093159 PMC7246680

[2] Melzer D , Pilling LC, Ferrucci L. The genetics of human ageing. Nat Rev Genet 2020; 21: 88–101. 31690828 10.1038/s41576-019-0183-6 PMC9934000

[3] Dhillon RJS , Hasni S. Pathogenesis and Management of Sarcopenia. Clin Geriatr Med 2017; 33: 17–26. 27886695 10.1016/j.cger.2016.08.002 PMC5127276

[4] Xia X , Jiang Q, McDermott J et al. Aging and Alzheimer’s disease: comparison and associations from molecular to system level. Aging Cell 2018; 17: e12802. 29963744 10.1111/acel.12802 PMC6156542

[5] Scarmeas N , Anastasiou CA, Yannakoulia M. Nutrition and prevention of cognitive impairment. Lancet Neurol 2018; 17: 1006–15. 30244829 10.1016/S1474-4422(18)30338-7

[6] Alasalvar C , Bolling BW. Review of nut phytochemicals, fat-soluble bioactives, antioxidant components and health effects. Br J Nutr 2015; 113: S68–78. 26148924 10.1017/S0007114514003729

[7] Hou Y , He W, Hu S et al. Composition of polyamines and amino acids in plant-source foods for human consumption. Amino Acids 2019; 51: 1153–65. 31197570 10.1007/s00726-019-02751-0

[8] Becerra-Tomás N , Paz-Graniel I, Kendall WC et al. Nut consumption and incidence of cardiovascular diseases and cardiovascular disease mortality: a meta-analysis of prospective cohort studies. Nutr Rev 2019; 77: 691–709. 31361320 10.1093/nutrit/nuz042 PMC6845198

[9] Shang X , Scott D, Hodge A et al. Dietary protein from different food sources, incident metabolic syndrome and changes in its components: an 11-year longitudinal study in healthy community-dwelling adults. Clin Nutr 2017; 36: 1540–8. 27746001 10.1016/j.clnu.2016.09.024

[10] Wu L , Wang Z, Zhu J et al. Nut consumption and risk of cancer and type 2 diabetes: a systematic review and meta-analysis. Nutr Rev 2015; 73: 409–25. 26081452 10.1093/nutrit/nuv006 PMC4560032

[11] Aune D , Keum N, Giovannucci E et al. Nut consumption and risk of cardiovascular disease, total cancer, all-cause and cause-specific mortality: a systematic review and dose-response meta-analysis of prospective studies. BMC Med 2016; 14: 207. 27916000 10.1186/s12916-016-0730-3 PMC5137221

[12] Li M , ShiZ. A prospective association of nut consumption with cognitive function in Chinese adults aged 55+ _ China health and nutrition survey. J Nutr Health Aging2019; 23: 211–6.30697633 10.1007/s12603-018-1122-5

[13] Bao Y , Han J, Hu FB et al. Association of nut consumption with Total and cause-specific mortality. N Engl J Med 2013; 369: 2001–11. 24256379 10.1056/NEJMoa1307352 PMC3931001

[14] Arias-Fernández L , Machado-Fragua MD, Graciani A et al. Prospective association between nut consumption and physical function in older men and women. J Gerontol Ser A. 2019; 74: 1091–7. 10.1093/gerona/gly171 30052782

[15] Theodore LE , Kellow NJ, McNeil EA et al. Nut consumption for cognitive performance: a systematic review. Adv Nutr 2021; 12: 777–92. 33330927 10.1093/advances/nmaa153 PMC8166568

[16] Witkowska AM , Waśkiewicz A, Zujko ME et al. The consumption of nuts is associated with better dietary and lifestyle patterns in polish adults: results of WOBASZ and WOBASZ II surveys. Nutrients 2019; 11: 1410. 31234530 10.3390/nu11061410 PMC6627533

[17] O’Neil C , Nicklas T, Iii V. Tree nut consumption is associated with better nutrient adequacy and diet quality in adults: National Health and nutrition examination survey 2005–2010. Nutrients 2015; 7: 595–607. 25599274 10.3390/nu7010595 PMC4303856

[18] Ward SJ , Hill AM, Buckley JD et al. Minimal changes in telomere length after a 12-week dietary intervention with almonds in mid-age to older, overweight and obese Australians: results of a randomised clinical trial. Br J Nutr 2022; 127: 872–84. 33971995 10.1017/S0007114521001549

[19] Tey SL , Brown R, Gray A et al. Nuts improve diet quality compared to other energy-dense snacks while maintaining body weight. J Nutr Metab 2011; 2011: 1–11. 10.1155/2011/357350 PMC3154486 21845219

[20] Wang W , Yang M, Kenfield SA et al. Nut consumption and prostate cancer risk and mortality. Br J Cancer 2016; 115: 371–4. 27280637 10.1038/bjc.2016.181 PMC4973153

[21] Study design of ASPirin in reducing events in the elderly (ASPREE): a randomized, controlled trial. Contemp Clin Trials 2013; 36: 555–64. 24113028 10.1016/j.cct.2013.09.014 PMC3919683

[22] McNeil JJ , Nelson MR, Woods RL et al. Effect of Aspirin on all-cause mortality in the healthy elderly. N Engl J Med 2018; 379: 1519–28. 30221595 10.1056/NEJMoa1803955 PMC6433466

[23] McNeil JJ , Woods RL, Ward SA et al. Cohort profile: the ASPREE longitudinal study of older persons (ALSOP). Int J Epidemiol 2019; 48: 1048–1049h. 30624660 10.1093/ije/dyy279 PMC6693806

[24] McNeil JJ , Woods RL, Nelson MR et al. Effect of Aspirin on disability-free survival in the healthy elderly. N Engl J Med 2018; 379: 1499–508. 30221596 10.1056/NEJMoa1800722 PMC6426126

[25] Woods RL , Espinoza S, Thao LTP et al. Effect of Aspirin on activities of daily living disability in community-dwelling older adults. Melzer D, editor. J Gerontol Ser A 2021; 76: 2007–14. 10.1093/gerona/glaa316 PMC8514067 33367621

[26] Australian Guidelines for Drinking Alcohol. Alcohol Think Again, Australia [Internet]. [cited 2023 Sep 6]; Available from: https://alcoholthinkagain.com.au/alcohol-and-your-health/alcohol-guidelines.

[27] Census of Population and Housing: Socio-Economic Indexes for Areas (SEIFA), Australian Bureau of Statistics. Australia, 2011 [Internet], [cited 2023 Sep 6]. Available from: https://www.abs.gov.au/ausstats/abs@.nsf/Lookup/2033.0.55.001main+features100042011.

[28] Ryan J , Espinoza S, Ernst ME et al. Validation of a deficit-accumulation frailty index in the ASPirin in reducing events in the elderly study and its predictive capacity for disability-free survival. Le Couteur D, editor. J Gerontol Ser A 2022; 77: 19–26. 10.1093/gerona/glab225 PMC8751791 34338761

[29] Radloff LS . The CES-D scale: a self-report depression scale for research in the general population. APM 1977; 1: 385–401.

[30] Dicken SJ , Batterham RL. The role of diet quality in mediating the association between ultra-processed food intake, obesity and health-related outcomes: a review of prospective cohort studies. Nutrients 2021; 14: 23. 35010898 10.3390/nu14010023 PMC8747015

[31] Yamakawa M , Wada K, Koda S et al. Associations of total nut and peanut intakes with all-cause and cause-specific mortality in a Japanese community: the Takayama study. Br J Nutr 2022; 127: 1378–85. 34225833 10.1017/S0007114521002257

[32] Liu G , Guasch-Ferré M, Hu Y et al. Nut consumption in relation to cardiovascular disease incidence and mortality among patients with diabetes mellitus. Circ Res 2019; 124: 920–9. 30776978 10.1161/CIRCRESAHA.118.314316 PMC6417933

[33] Cahoon D , Shertukde SP, Avendano EE et al. Walnut intake, cognitive outcomes and risk factors: a systematic review and meta-analysis. Ann Med 2021; 53: 972–98. 10.1080/07853890.2021.1925955 PMC8211141 34132152

[34] O’Brien J , Okereke O, Devore E et al. Long-term intake of nuts in relation to cognitive function in older women. J Nutr Health Aging 2014; 18: 496–502. 24886736 10.1007/s12603-014-0014-6 PMC4105147

[35] Xu X , Inglis SC, Parker D. Sex differences in dietary consumption and its association with frailty among middle-aged and older Australians: a 10-year longitudinal survey. BMC Geriatr 2021; 21: 217. 33789566 10.1186/s12877-021-02165-2 PMC8011098

[36] Coelho-Junior HJ , Calvani R, Picca A et al. Protein intake and frailty in older adults: a systematic review and meta-analysis of observational studies. Nutrients 2022; 14: 2767. 36432568 10.3390/nu14224881 PMC9698248

[37] Wang R , Hannan MT, Wang M et al. Long-term consumption of nuts (including peanuts, peanut butter, walnuts, and other nuts) in relation to risk of frailty in older women: evidence from a cohort study. J Nutr 2023; 153: 820–7. 36931754 10.1016/j.tjnut.2023.01.003 PMC10196568

[38] Nutrient Reference Values for Australia and New Zealand Including Recommended Dietary Intakes. NHMRC, Australia [Internet]. [cited 2023 Sep 6]; Available from: https://www.nhmrc.gov.au/about-us/publications/nutrient-reference-values-australia-and-new-zealand-including-recommended-dietary-intakes#block-views-block-file-attachments-content-block-1.

[39] Tan SY , Tey S, Brown R. Can nuts mitigate malnutrition in older adults? A conceptual framework. Nutrients 2018; 10: 1448. 30301198 10.3390/nu10101448 PMC6213172

[40] Sabaté J , Oda K, Ros E. Nut consumption and blood lipid levels: a pooled analysis of 25 intervention trials. Arch Intern Med 2010; 170: 821–7. 20458092 10.1001/archinternmed.2010.79

[41] Del Gobbo LC , Falk MC, Feldman R et al. Effects of tree nuts on blood lipids, apolipoproteins, and blood pressure: systematic review, meta-analysis, and dose-response of 61 controlled intervention trials. Am J Clin Nutr 2015; 102: 1347–56. 26561616 10.3945/ajcn.115.110965 PMC4658458

[42] Xiao Y , Huang W, Peng C et al. Effect of nut consumption on vascular endothelial function: a systematic review and meta-analysis of randomized controlled trials. Clin Nutr 2018; 37: 831–9. 28457654 10.1016/j.clnu.2017.04.011

[43] Ren J , Chung SH. Anti-inflammatory effect of α-linolenic acid and its mode of action through the inhibition of nitric oxide production and inducible nitric oxide synthase gene expression via NF-κB and mitogen-activated protein kinase pathways. J Agric Food Chem 2007; 55: 5073–80. 17542608 10.1021/jf0702693

[44] Cutuli D . Functional and structural benefits induced by omega-3 polyunsaturated fatty acids during aging. Curr Neuropharmacol 2017; 15: 534–42. 27306037 10.2174/1570159X14666160614091311 PMC5543674

[45] Geravand S , Karami M, Sahraei H et al. Protective effects of L-arginine on Alzheimer’s disease: modulating hippocampal nitric oxide levels and memory deficits in aluminum chloride-induced rat model. Eur J Pharmacol 2023; 958: 176030. 37660966 10.1016/j.ejphar.2023.176030

[46] Salas-Salvadó J , Bulló M, Pérez-Heras A et al. Dietary fibre, nuts and cardiovascular diseases. Br J Nutr 2006; 96: S45–51. 10.1017/bjn20061863 17125533

[47] Kim Y , Keogh JB, Clifton PM. Benefits of nut consumption on insulin resistance and cardiovascular risk factors: multiple potential mechanisms of actions. Nutrients 2017; 9: 1271. 29165404 10.3390/nu9111271 PMC5707743

[48] Mead LC , Hill AM, Carter S et al. The effect of nut consumption on diet quality, Cardiometabolic and gastrointestinal health in children: a systematic review of randomized controlled trials. Int J Environ Res Public Health 2021; 18: 454. 33430029 10.3390/ijerph18020454 PMC7827804

[49] Rehm CD , Drewnowski A. Replacing American snacks with tree nuts increases consumption of key nutrients among US children and adults: results of an NHANES modeling study. Nutr J 2017; 16: 17. 28270158 10.1186/s12937-017-0238-5 PMC5341477

[50] Meddens SFW , de Vlaming R, Bowers P et al. Genomic analysis of diet composition finds novel loci and associations with health and lifestyle. Mol Psychiatry 2021; 26: 2056–69. 32393786 10.1038/s41380-020-0697-5 PMC7767645

