# Supplementary material for: Nut consumption and disability-free survival in community-dwelling older adults: a prospective cohort study
Source: Age Ageing. 2024 Nov 18;53(11):afae239. doi: 10.1093/ageing/afae239 (PMC11570366; doi:10.1093/ageing/afae239)

**Nut consumption and disability-free survival in community-dwelling older adults: A prospective cohort study.**

**Appendix 1. Food Frequency Questionnaire**


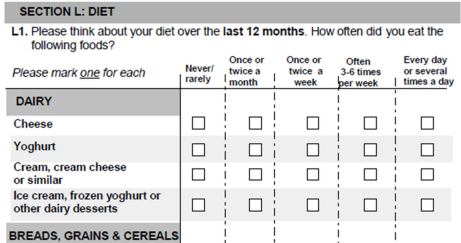

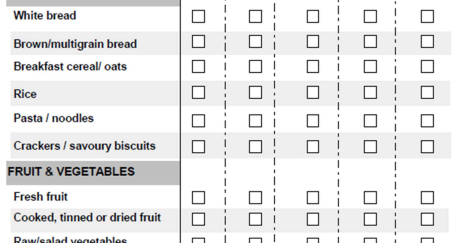

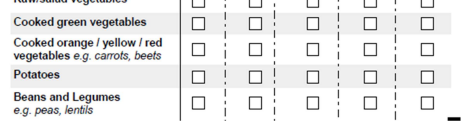


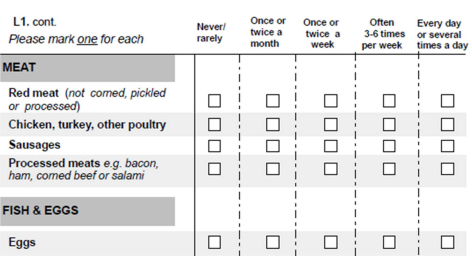

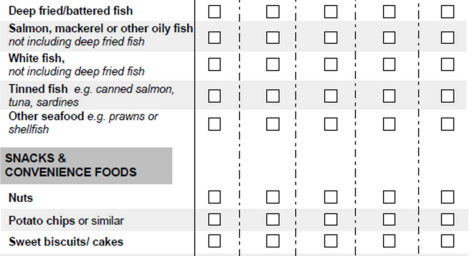

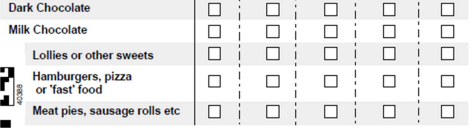


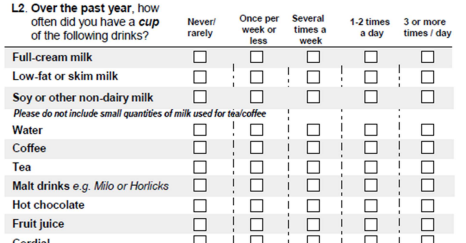

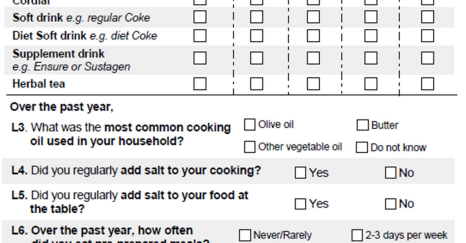

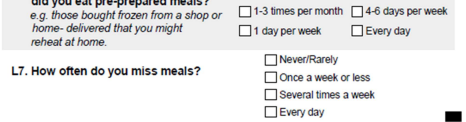

Supplement: aa-24-0477-File002_afae239 [file aa-24-0477-file002_afae239.docx]
