# Supplementary material for: Nut consumption and disability-free survival in community-dwelling older adults: a prospective cohort study
Source: Age Ageing. 2024 Nov 18;53(11):afae239. doi: 10.1093/ageing/afae239 (PMC11570366; doi:10.1093/ageing/afae239)
Supplement: aa-24-0477-File003_afae239 [file aa-24-0477-file003_afae239.docx]

**Nut consumption and disability-free survival in community-dwelling older adults: A prospective cohort study.**

**Appendix 2.**

**Sensitivity Analysis Methods**

To understand the influence of overall protein consumption on the association between nut intake and disability-free survival a sensitivity analysis was conducted. The total protein score was created using participant responses to FFQ questions the frequency of consumption of protein-rich food sources: meat (red meat, poultry), fish (tinned, white, oily, fried), eggs, dairy products (yogurt, milk, cream, cheese), and legume. Aligning with previous research, grains were not included as a protein source in our study. (1, 2, 3) Participants were asked to report the frequency of consumption of the above foods (rarely/never; once/twice a month; once/twice a week; 3-6 times a week; daily/several times daily) and beverages (once per week. A binary score was created for each food, representing weekly consumption, 0 (rarely/never; once/twice a month) or 1 (once/twice a week; 3-6 times a week; daily/several times daily). These binary scores were then added together to create a diversity score (0-14) that represented the weekly consumption of protein sources. Cox proportional hazards regression analysis was used to assess the association between nut intake and disability-free survival. A crude model was performed followed; a minimally adjusted model [age and sex], and a fully adjusted model [age, sex IRSAD quintile and education, physical capacity, smoking status and alcohol consumption, waist circumference, hypertension, type 2 diabetes, depression score, frailty status, and self-reported oral health status, total protein score and dietary quality score].

**Table 1. Subgroup Analysis:** *Cox proportional hazards regression analysis of the association between nut consumption and disability free survival, stratified by dietary quality score tertile.*

| Crude Model | Dietary Quality Score Tertiles | | |
| --- | --- | --- | --- |
|  | T1 | T2 | T3 |
| Infrequent /Never  Weekly  Daily | Ref  0.96 [0.50-1.87]. 0.916  - | Ref  0.83 [0.70-0.97], 0.020  0.64 [0.47-0.89]. 0.008 | Ref  0.82 [0.62-.1.10], 0.199  0.80 [0.56-1.15], 0.231 |
| Minimally Adjusted Multivariate Model ^a^ |  |  |  |
| Infrequent  Weekly  Daily | Ref  1.02 [.0.53-2.0]. 0.936  - | Ref  0.88 [0.75-1.04], 0.134  0.66 [0.48-0.92]. 0.013 | Ref  0.87 [0.66-.1.17], 0.383  0.81 [0.56-1.17], 0.268 |
| Fully Adjusted Multivariate Model ^b^ |  |  |  |
| Infrequent  Weekly  Daily | Ref  1.10 [0.54-2.18], 0.810  - | Ref  0.93 [0.79-1.10], 0.365  0.71 [0.51-0.98]. 0.037 | Ref  0.95 [0.71-.1.30], 0.733  0.86 [0.60-1.24], 0.418 |

**^a^ Minimally Adjusted Model:** Adjusted for Age and Sex

**^b^ Fully Adjusted Model:** Adjusted for IRSAD, education physical ability, smoking status, alcohol consumption, waist circumference hypertension, type 2 diabetes, depression (CES-D-10), frailty score, self-reported oral health & diet quality score tertile.

**Table 2. Sensitivity Analysis:** *Cox Proportional Hazards Regression Analysis of the association between nut consumption and Disability Free Survival, controlled for overall protein intake.*

| Crude Model | |
| --- | --- |
| Nut Consumption | HR [95%CI’s], p-value |
| Infrequent  Weekly  Daily | Ref  0.79 [0.69-0.91], 0.001  0.65 [0.52-0.82]. <0.001 |
| Minimally Adjusted Multivariate Model ^a^ | |
| Infrequent  Weekly  Daily | Ref  0.86 [0.75-0.99] 0.026  0.68 [0.55-0.90], 0.001 |
| Fully Adjusted Multivariate Model b | |
| Infrequent  Weekly  Daily | Ref  0.93 [0.81-1.07], 0.356  0.76 [0.60-0.95], 0.030 |

**^a^ Minimally Adjusted Model:** Adjusted for Age and Sex

**^b^ Fully Adjusted Model:** Adjusted for IRSAD, education physical ability, smoking status, alcohol consumption, waist circumference hypertension, type 2 diabetes , depression (CES-D-10), frailty score, self-reported oral health, overall protein intake, dietary quality score

**References**

1 Xue Q, Shen M, Lin Q, Wu X, Yang M. The Association between Dietary Protein Diversity and Protein Patterns with Frailty in older Chinese adults: a Population-based Cohort Study. J Nutr Health Aging. 2023;27(12):1219–27.

2 Yin Z, Fei Z, Qiu C, Brasher MS, Kraus VB, Zhao W, et al. Dietary diversity and cognitive function among elderly people: A population-based study. J Nutr Health Aging. 2017;21:1089–94.

3 Wang XM, Zhong WF, Li ZH, Chen PL, Zhang YJ, Ren JJ, et al. Dietary diversity and frailty among older Chinese people: evidence from the Chinese Longitudinal Healthy Longevity Study. Am J Clin Nutr. 2023;117(2):383–91.
